# Dimethyl 2,6-dimethyl-4-(3-phenyl-1*H*-pyrazol-4-yl)-1,4-dihydro­pyridine-3,5-dicarboxyl­ate

**DOI:** 10.1107/S1600536812008306

**Published:** 2012-03-03

**Authors:** Hoong-Kun Fun, Suhana Arshad, Shridhar Malladi, Kammasandra Nanjunda Shivananda, Arun M. Isloor

**Affiliations:** a X-ray Crystallography Unit, School of Physics, Universiti Sains Malaysia, 11800 USM, Penang, Malaysia; b Medicinal Chemistry Division, Department of Chemistry, National Institute of Technology-Karnataka, Surathkal, Mangalore 575 025, India; c Schulich Faculty of Chemistry, Technion Israel Institute of Technology, Haifa 32000, Israel

## Abstract

In the title compound, C_20_H_21_N_3_O_4_, the 1,4-dihydro­pyridine ring adopts a boat conformation. An intra­molecular C—H⋯O hydrogen bond generates an *S*(6) ring motif. The pyrazole ring makes dihedral angles of 87.81 (7) and 45.09 (7)° with the mean plane of the 1,4-dihydro­pyridine ring and the phenyl ring, respectively. In the crystal, mol­ecules are linked by N—H⋯N, N—H⋯O and C—H⋯O hydrogen bonds into a three-dimensional network.

## Related literature

For a related structure and background references, see: Fun *et al.* (2011 ▶). For hydrogen-bond motifs, see: Bernstein *et al.* (1995 ▶). For the stability of the temperature controller used for data collection, see: Cosier & Glazer (1986 ▶).
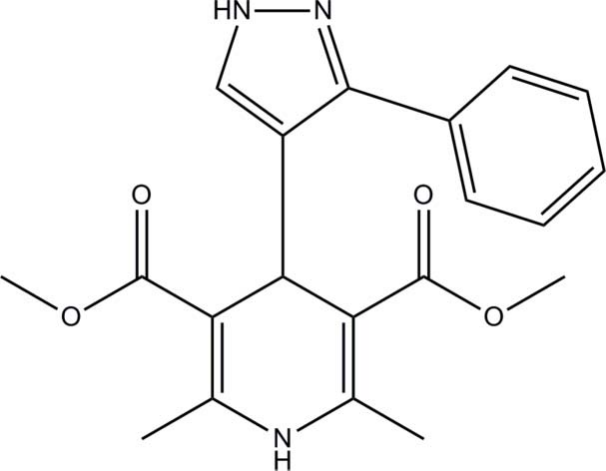


## Experimental

### 

#### Crystal data

C_20_H_21_N_3_O_4_
*M* *_r_* = 367.40
Orthorhombic, 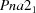

*a* = 13.9632 (6) Å
*b* = 10.9908 (5) Å
*c* = 11.8465 (5) Å
*V* = 1818.04 (14) Å^3^
*Z* = 4
Mo *K*α radiation
μ = 0.10 mm^−1^
*T* = 100 K
0.38 × 0.22 × 0.14 mm

#### Data collection

Bruker SMART APEXII DUO CCD diffractometer
Absorption correction: multi-scan (*SADABS*; Bruker, 2009 ▶) *T*_min_ = 0.965, *T*_max_ = 0.987
17004 measured reflections
2788 independent reflections
2687 reflections with *I* > 2σ(*I*)
*R*_int_ = 0.030

#### Refinement

*R*[*F* ^2^ > 2σ(*F* ^2^)] = 0.030
*wR*(*F* ^2^) = 0.080
*S* = 1.03
2788 reflections
256 parameters
1 restraint
H atoms treated by a mixture of independent and constrained refinement
Δρ_max_ = 0.30 e Å^−3^
Δρ_min_ = −0.24 e Å^−3^

### 

Data collection: *APEX2* (Bruker, 2009 ▶); cell refinement: *SAINT* (Bruker, 2009 ▶); data reduction: *SAINT*; program(s) used to solve structure: *SHELXTL* (Sheldrick, 2008 ▶); program(s) used to refine structure: *SHELXTL*; molecular graphics: *SHELXTL*; software used to prepare material for publication: *SHELXTL* and *PLATON* (Spek, 2009 ▶).

## Supplementary Material

Crystal structure: contains datablock(s) global, I. DOI: 10.1107/S1600536812008306/hb6648sup1.cif

Structure factors: contains datablock(s) I. DOI: 10.1107/S1600536812008306/hb6648Isup2.hkl

Supplementary material file. DOI: 10.1107/S1600536812008306/hb6648Isup3.cml

Additional supplementary materials:  crystallographic information; 3D view; checkCIF report

## Figures and Tables

**Table 1:** Hydrogen-bond geometry (Å, °)

*D*—H⋯*A*	*D*—H	H⋯*A*	*D*⋯*A*	*D*—H⋯*A*
C18—H18*A*⋯O3	0.98	2.29	2.9114 (18)	121
N3—H1N3⋯N2^i^	0.93 (2)	2.14 (2)	3.0529 (17)	167 (2)
N1—H1N1⋯O3^ii^	0.84 (3)	2.01 (3)	2.8438 (16)	173 (2)
C5—H5*A*⋯O1^iii^	0.95	2.60	3.4895 (18)	157
C20—H20*C*⋯O1^iv^	0.98	2.38	3.3524 (19)	170

Symmetry codes: (i) 
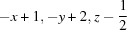
; (ii) 
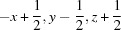
; (iii) 
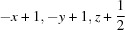
; (iv) 
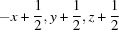
.

**Fig. 1.**
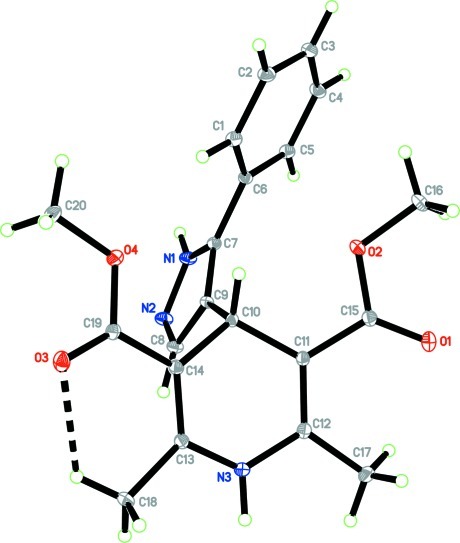
The molecular structure of the title compound with 30% probability displacement ellipsoids.

**Fig. 2.**
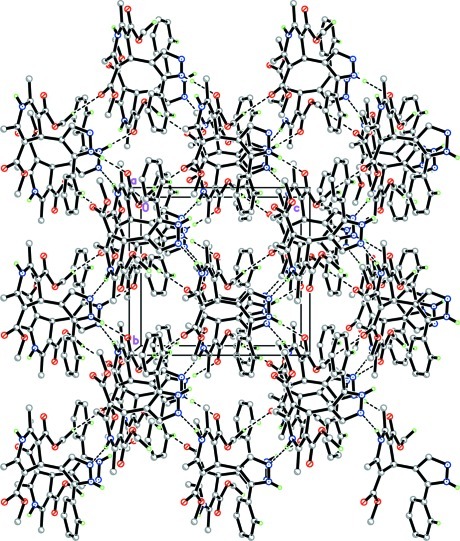
The crystal packing of the title compound. The H atoms not involved in the intermolecular interactions (dashed lines) have been omitted for clarity.

## supplementary crystallographic information

### Comment

As part of our ongoing studies of dihydropyridine/pyrazole derivatives, we have synthesized the title compound, (I), to study its crystal structure.

The molecular structure in shown in Fig. 1. The 1,4-dihydropyridine ring (N3/C10–C14) adopts a boat conformation with puckering parameters Q = 0.3273 (13) Å, Θ= 106.1 (2)° and Φ= 356.4 (2)°. An intramolecular C18—H18A···O3 hydrogen bond (Table 1) forms an *S*(6) ring motif (Bernstein *et al.*, 1995). The 1*H*-pyrazole ring (N1/N2/C7–C9) makes dihedral angles of 87.81 (7) and 45.09 (7)° with the mean plane of the 1,4-dihydropyridine (N3/C10–C14) ring and benzene (C1–C6) ring, respectively. Bond lengths and angles are comparable to the related structure (Fun *et al.*, 2011).

In the crystal structure (Fig. 2), the molecules are linked *via* intermolecular N3—H1N3···N2, N1—H1N1···O3, C5—H5A···O1 and C20—H20C···O1 hydrogen bonds (Table 1) into three-dimensional network.

### Experimental

3-Phenyl-1*H*-pyrazole-4-carbaldehyde (0.172 g, 1.0 mmol), methylacetoacetate (0.232 g, 2.0 mmol) and ammonium acetate (0.092 g, 1.2 mmol) in ethanol (7 ml) were refluxed for 5 h. After the completion of the reaction, the reaction mixture was concentrated and poured into crushed ice. The precipitated product was filtered and washed with water. The resulting solid was recrystallized from ethanol:water mixture as yellow blocks. Yield: 0.28 g, 76.21%. *M.p.*: 398–400 K.

### Refinement

N-bound H atoms was located from the difference map and refined freely, [N–H = 0.84 (3) and 0.93 (2) Å]. All the other H atoms were positioned geometrically [C–H = 0.95–1.00 Å] and refined using a riding model with *U*_iso_(H) = 1.2 or 1.5 *U*_eq_(C). A rotating group model was applied to the methyl groups. Ten outliners 8 3 5, 6 0 - 7, 8 0 6, 8 1 6, 8 2 5, 8 1 5, 8 0 5, 6 0 4, 4 0 3 and 8 5 0 were omitted. Since there are not sufficent anomalous dispersion to determine the absolute structure, 2358 Freidel pairs were merged for the final refinement.

### Crystal data

**Table:**

C_20_H_21_N_3_O_4_	*F*(000) = 776
*M**_r_* = 367.40	*D*_x_ = 1.342 Mg m^−^^3^
Orthorhombic, *P**n**a*2_1_	Mo *K*α radiation, λ = 0.71073 Å
Hall symbol: P 2c -2n	Cell parameters from 8800 reflections
*a* = 13.9632 (6) Å	θ = 2.4–30.1°
*b* = 10.9908 (5) Å	µ = 0.10 mm^−^^1^
*c* = 11.8465 (5) Å	*T* = 100 K
*V* = 1818.04 (14) Å^3^	Block, yellow
*Z* = 4	0.38 × 0.22 × 0.14 mm

### Data collection

**Table:**

Bruker SMART APEXII DUO CCD diffractometer	2788 independent reflections
Radiation source: fine-focus sealed tube	2687 reflections with *I* > 2σ(*I*)
Graphite monochromator	*R*_int_ = 0.030
φ and ω scans	θ_max_ = 30.1°, θ_min_ = 2.4°
Absorption correction: multi-scan (*SADABS*; Bruker, 2009)	*h* = −19→19
*T*_min_ = 0.965, *T*_max_ = 0.987	*k* = −14→15
17004 measured reflections	*l* = −16→15

### Refinement

**Table:**

Refinement on *F*^2^	Primary atom site location: structure-invariant direct methods
Least-squares matrix: full	Secondary atom site location: difference Fourier map
*R*[*F*^2^ > 2σ(*F*^2^)] = 0.030	Hydrogen site location: inferred from neighbouring sites
*wR*(*F*^2^) = 0.080	H atoms treated by a mixture of independent and constrained refinement
*S* = 1.03	*w* = 1/[σ^2^(*F*_o_^2^) + (0.0556*P*)^2^ + 0.1941*P*] where *P* = (*F*_o_^2^ + 2*F*_c_^2^)/3
2788 reflections	(Δ/σ)_max_ = 0.001
256 parameters	Δρ_max_ = 0.30 e Å^−^^3^
1 restraint	Δρ_min_ = −0.24 e Å^−^^3^

### Special details

**Table:**

Experimental. The crystal was placed in the cold stream of an Oxford Cryosystems Cobra open-flow nitrogen cryostat (Cosier & Glazer, 1986) operating at 100.0 (1) K.
Geometry. All e.s.d.'s (except the e.s.d. in the dihedral angle between two l.s. planes) are estimated using the full covariance matrix. The cell e.s.d.'s are taken into account individually in the estimation of e.s.d.'s in distances, angles and torsion angles; correlations between e.s.d.'s in cell parameters are only used when they are defined by crystal symmetry. An approximate (isotropic) treatment of cell e.s.d.'s is used for estimating e.s.d.'s involving l.s. planes.
Refinement. Refinement of *F*^2^ against ALL reflections. The weighted *R*-factor *wR* and goodness of fit *S* are based on *F*^2^, conventional *R*-factors *R* are based on *F*, with *F* set to zero for negative *F*^2^. The threshold expression of *F*^2^ > σ(*F*^2^) is used only for calculating *R*-factors(gt) *etc*. and is not relevant to the choice of reflections for refinement. *R*-factors based on *F*^2^ are statistically about twice as large as those based on *F*, and *R*- factors based on ALL data will be even larger.

### Fractional atomic coordinates and isotropic or equivalent isotropic displacement parameters (Å^2^)

**Table:**

	*x*	*y*	*z*	*U*_iso_*/*U*_eq_	
O1	0.46234 (8)	0.61784 (10)	0.30293 (11)	0.0235 (2)	
O2	0.32205 (8)	0.59619 (9)	0.39414 (10)	0.0205 (2)	
O3	0.15716 (8)	1.09597 (9)	0.48010 (9)	0.0191 (2)	
O4	0.14156 (7)	0.90479 (9)	0.54372 (10)	0.0178 (2)	
N1	0.37507 (9)	0.72586 (11)	0.77599 (10)	0.0156 (2)	
N2	0.42080 (9)	0.83459 (11)	0.77451 (10)	0.0175 (2)	
N3	0.44724 (8)	0.99042 (10)	0.40046 (10)	0.0142 (2)	
C1	0.18445 (9)	0.58580 (12)	0.61291 (12)	0.0156 (2)	
H1A	0.1551	0.6602	0.5914	0.019*	
C2	0.13659 (10)	0.47622 (13)	0.59534 (13)	0.0181 (3)	
H2A	0.0751	0.4764	0.5611	0.022*	
C3	0.17821 (10)	0.36647 (13)	0.62751 (13)	0.0186 (3)	
H3A	0.1454	0.2919	0.6152	0.022*	
C4	0.26818 (10)	0.36682 (12)	0.67776 (12)	0.0184 (3)	
H4A	0.2966	0.2922	0.7004	0.022*	
C5	0.31710 (10)	0.47597 (12)	0.69520 (12)	0.0154 (2)	
H5A	0.3786	0.4753	0.7295	0.018*	
C6	0.27552 (9)	0.58674 (11)	0.66210 (11)	0.0130 (2)	
C7	0.32755 (9)	0.70190 (11)	0.67790 (11)	0.0123 (2)	
C8	0.40266 (10)	0.87859 (12)	0.67155 (12)	0.0155 (2)	
H8A	0.4266	0.9542	0.6448	0.019*	
C9	0.34428 (9)	0.80077 (11)	0.60694 (11)	0.0120 (2)	
C10	0.31482 (9)	0.82217 (11)	0.48536 (11)	0.0114 (2)	
H10A	0.2572	0.7714	0.4684	0.014*	
C11	0.39518 (9)	0.78588 (11)	0.40562 (11)	0.0122 (2)	
C12	0.46244 (9)	0.86881 (12)	0.37489 (11)	0.0136 (2)	
C13	0.35839 (9)	1.03464 (11)	0.43207 (11)	0.0128 (2)	
C14	0.28952 (9)	0.95537 (11)	0.46742 (11)	0.0126 (2)	
C15	0.39982 (10)	0.66120 (12)	0.36172 (12)	0.0147 (2)	
C16	0.31848 (13)	0.47237 (13)	0.35474 (17)	0.0275 (3)	
H16A	0.2655	0.4297	0.3918	0.041*	
H16B	0.3790	0.4315	0.3727	0.041*	
H16C	0.3085	0.4717	0.2729	0.041*	
C17	0.55479 (10)	0.84310 (13)	0.31417 (14)	0.0191 (3)	
H17A	0.5858	0.7719	0.3480	0.029*	
H17B	0.5973	0.9137	0.3205	0.029*	
H17C	0.5415	0.8269	0.2344	0.029*	
C18	0.35149 (10)	1.17080 (12)	0.42430 (12)	0.0173 (2)	
H18A	0.2991	1.1995	0.4724	0.026*	
H18B	0.3390	1.1944	0.3459	0.026*	
H18C	0.4119	1.2072	0.4495	0.026*	
C19	0.19231 (9)	0.99557 (12)	0.49572 (11)	0.0134 (2)	
C20	0.04439 (10)	0.93544 (15)	0.57625 (14)	0.0215 (3)	
H20A	0.0114	0.8621	0.6027	0.032*	
H20B	0.0102	0.9690	0.5110	0.032*	
H20C	0.0458	0.9960	0.6370	0.032*	
H1N3	0.4908 (15)	1.047 (2)	0.373 (2)	0.025 (5)*	
H1N1	0.3705 (16)	0.689 (2)	0.838 (2)	0.032 (6)*	

### Atomic displacement parameters (Å^2^)

**Table:**

	*U*^11^	*U*^22^	*U*^33^	*U*^12^	*U*^13^	*U*^23^
O1	0.0253 (5)	0.0200 (5)	0.0252 (5)	0.0015 (4)	0.0071 (4)	−0.0072 (4)
O2	0.0264 (5)	0.0108 (4)	0.0242 (5)	−0.0030 (4)	0.0078 (4)	−0.0029 (4)
O3	0.0224 (5)	0.0171 (5)	0.0176 (5)	0.0067 (4)	0.0022 (4)	0.0028 (4)
O4	0.0129 (4)	0.0163 (4)	0.0243 (5)	−0.0002 (3)	0.0041 (4)	−0.0003 (4)
N1	0.0178 (5)	0.0164 (5)	0.0126 (5)	−0.0043 (4)	−0.0023 (4)	0.0031 (4)
N2	0.0193 (5)	0.0176 (5)	0.0156 (5)	−0.0068 (4)	−0.0020 (4)	0.0010 (4)
N3	0.0147 (5)	0.0125 (5)	0.0153 (5)	−0.0017 (4)	0.0018 (4)	0.0008 (4)
C1	0.0145 (6)	0.0155 (6)	0.0168 (6)	0.0001 (4)	−0.0005 (5)	0.0018 (5)
C2	0.0140 (6)	0.0207 (6)	0.0197 (6)	−0.0030 (5)	−0.0012 (5)	−0.0009 (5)
C3	0.0208 (7)	0.0160 (6)	0.0190 (6)	−0.0044 (5)	0.0025 (5)	−0.0021 (5)
C4	0.0225 (7)	0.0125 (5)	0.0201 (6)	−0.0004 (5)	0.0003 (5)	0.0019 (5)
C5	0.0153 (6)	0.0150 (5)	0.0157 (6)	0.0000 (4)	−0.0008 (4)	0.0022 (5)
C6	0.0139 (5)	0.0129 (5)	0.0122 (5)	−0.0014 (4)	0.0005 (4)	0.0009 (4)
C7	0.0112 (5)	0.0128 (5)	0.0129 (5)	−0.0004 (4)	−0.0005 (4)	0.0011 (4)
C8	0.0169 (6)	0.0150 (5)	0.0146 (6)	−0.0045 (4)	−0.0009 (5)	0.0006 (5)
C9	0.0127 (5)	0.0109 (5)	0.0124 (5)	−0.0008 (4)	0.0007 (4)	0.0004 (4)
C10	0.0127 (5)	0.0104 (5)	0.0111 (5)	0.0011 (4)	0.0007 (4)	0.0002 (4)
C11	0.0144 (5)	0.0115 (5)	0.0107 (5)	0.0016 (4)	0.0006 (4)	−0.0003 (4)
C12	0.0150 (5)	0.0140 (5)	0.0120 (5)	0.0022 (4)	0.0001 (4)	0.0008 (4)
C13	0.0159 (6)	0.0118 (5)	0.0109 (5)	0.0001 (4)	−0.0002 (4)	0.0005 (4)
C14	0.0144 (5)	0.0114 (5)	0.0120 (5)	0.0009 (4)	0.0003 (4)	0.0013 (4)
C15	0.0182 (6)	0.0130 (5)	0.0130 (5)	0.0010 (4)	−0.0002 (5)	0.0010 (4)
C16	0.0344 (9)	0.0131 (6)	0.0350 (9)	−0.0061 (6)	0.0101 (7)	−0.0078 (6)
C17	0.0171 (6)	0.0190 (6)	0.0211 (6)	0.0014 (5)	0.0059 (5)	0.0008 (5)
C18	0.0214 (6)	0.0107 (5)	0.0199 (6)	−0.0008 (4)	0.0021 (5)	0.0017 (5)
C19	0.0149 (5)	0.0149 (5)	0.0103 (5)	0.0004 (4)	−0.0001 (4)	−0.0009 (4)
C20	0.0124 (6)	0.0252 (7)	0.0270 (7)	−0.0012 (5)	0.0041 (5)	−0.0059 (6)

### Geometric parameters (Å, º)

**Table:**

O1—C15	1.2142 (17)	C7—C9	1.3936 (18)
O2—C15	1.3555 (17)	C8—C9	1.4078 (18)
O2—C16	1.4396 (17)	C8—H8A	0.9500
O3—C19	1.2219 (16)	C9—C10	1.5163 (18)
O4—C19	1.3494 (16)	C10—C11	1.5200 (17)
O4—C20	1.4501 (16)	C10—C14	1.5209 (17)
N1—N2	1.3551 (16)	C10—H10A	1.0000
N1—C7	1.3638 (16)	C11—C12	1.3584 (17)
N1—H1N1	0.84 (3)	C11—C15	1.4671 (17)
N2—C8	1.3363 (18)	C12—C17	1.5034 (18)
N3—C13	1.3840 (17)	C13—C14	1.3635 (17)
N3—C12	1.3868 (16)	C13—C18	1.5024 (17)
N3—H1N3	0.93 (2)	C14—C19	1.4663 (18)
C1—C2	1.3929 (19)	C16—H16A	0.9800
C1—C6	1.3989 (18)	C16—H16B	0.9800
C1—H1A	0.9500	C16—H16C	0.9800
C2—C3	1.392 (2)	C17—H17A	0.9800
C2—H2A	0.9500	C17—H17B	0.9800
C3—C4	1.390 (2)	C17—H17C	0.9800
C3—H3A	0.9500	C18—H18A	0.9800
C4—C5	1.3958 (18)	C18—H18B	0.9800
C4—H4A	0.9500	C18—H18C	0.9800
C5—C6	1.4045 (17)	C20—H20A	0.9800
C5—H5A	0.9500	C20—H20B	0.9800
C6—C7	1.4713 (17)	C20—H20C	0.9800
			
C15—O2—C16	115.74 (12)	C12—C11—C15	120.08 (11)
C19—O4—C20	115.57 (11)	C12—C11—C10	120.06 (11)
N2—N1—C7	112.86 (11)	C15—C11—C10	119.87 (11)
N2—N1—H1N1	117.8 (16)	C11—C12—N3	118.84 (11)
C7—N1—H1N1	128.1 (16)	C11—C12—C17	126.52 (12)
C8—N2—N1	103.98 (11)	N3—C12—C17	114.64 (11)
C13—N3—C12	122.32 (11)	C14—C13—N3	119.40 (11)
C13—N3—H1N3	116.5 (13)	C14—C13—C18	127.59 (12)
C12—N3—H1N3	117.8 (13)	N3—C13—C18	112.99 (11)
C2—C1—C6	120.31 (12)	C13—C14—C19	122.05 (11)
C2—C1—H1A	119.8	C13—C14—C10	119.61 (11)
C6—C1—H1A	119.8	C19—C14—C10	118.23 (11)
C3—C2—C1	120.53 (13)	O1—C15—O2	122.11 (12)
C3—C2—H2A	119.7	O1—C15—C11	126.99 (13)
C1—C2—H2A	119.7	O2—C15—C11	110.89 (11)
C4—C3—C2	119.47 (13)	O2—C16—H16A	109.5
C4—C3—H3A	120.3	O2—C16—H16B	109.5
C2—C3—H3A	120.3	H16A—C16—H16B	109.5
C3—C4—C5	120.53 (13)	O2—C16—H16C	109.5
C3—C4—H4A	119.7	H16A—C16—H16C	109.5
C5—C4—H4A	119.7	H16B—C16—H16C	109.5
C4—C5—C6	120.09 (13)	C12—C17—H17A	109.5
C4—C5—H5A	120.0	C12—C17—H17B	109.5
C6—C5—H5A	120.0	H17A—C17—H17B	109.5
C1—C6—C5	119.05 (11)	C12—C17—H17C	109.5
C1—C6—C7	120.54 (11)	H17A—C17—H17C	109.5
C5—C6—C7	120.40 (12)	H17B—C17—H17C	109.5
N1—C7—C9	106.37 (11)	C13—C18—H18A	109.5
N1—C7—C6	120.98 (11)	C13—C18—H18B	109.5
C9—C7—C6	132.60 (12)	H18A—C18—H18B	109.5
N2—C8—C9	112.72 (12)	C13—C18—H18C	109.5
N2—C8—H8A	123.6	H18A—C18—H18C	109.5
C9—C8—H8A	123.6	H18B—C18—H18C	109.5
C7—C9—C8	104.06 (12)	O3—C19—O4	121.37 (12)
C7—C9—C10	130.43 (12)	O3—C19—C14	127.54 (12)
C8—C9—C10	125.40 (12)	O4—C19—C14	111.09 (11)
C9—C10—C11	110.45 (10)	O4—C20—H20A	109.5
C9—C10—C14	110.18 (10)	O4—C20—H20B	109.5
C11—C10—C14	109.71 (10)	H20A—C20—H20B	109.5
C9—C10—H10A	108.8	O4—C20—H20C	109.5
C11—C10—H10A	108.8	H20A—C20—H20C	109.5
C14—C10—H10A	108.8	H20B—C20—H20C	109.5
			
C7—N1—N2—C8	−1.15 (16)	C14—C10—C11—C15	−147.94 (11)
C6—C1—C2—C3	0.7 (2)	C15—C11—C12—N3	168.44 (12)
C1—C2—C3—C4	0.1 (2)	C10—C11—C12—N3	−11.42 (18)
C2—C3—C4—C5	−0.5 (2)	C15—C11—C12—C17	−11.6 (2)
C3—C4—C5—C6	0.1 (2)	C10—C11—C12—C17	168.57 (13)
C2—C1—C6—C5	−1.1 (2)	C13—N3—C12—C11	−15.16 (19)
C2—C1—C6—C7	178.25 (13)	C13—N3—C12—C17	164.85 (12)
C4—C5—C6—C1	0.7 (2)	C12—N3—C13—C14	17.20 (19)
C4—C5—C6—C7	−178.63 (13)	C12—N3—C13—C18	−163.94 (12)
N2—N1—C7—C9	0.79 (15)	N3—C13—C14—C19	−176.37 (12)
N2—N1—C7—C6	178.43 (12)	C18—C13—C14—C19	5.0 (2)
C1—C6—C7—N1	136.67 (13)	N3—C13—C14—C10	7.47 (18)
C5—C6—C7—N1	−43.99 (18)	C18—C13—C14—C10	−171.21 (13)
C1—C6—C7—C9	−46.4 (2)	C9—C10—C14—C13	92.04 (14)
C5—C6—C7—C9	132.93 (16)	C11—C10—C14—C13	−29.76 (16)
N1—N2—C8—C9	1.08 (16)	C9—C10—C14—C19	−84.27 (14)
N1—C7—C9—C8	−0.10 (14)	C11—C10—C14—C19	153.92 (11)
C6—C7—C9—C8	−177.35 (13)	C16—O2—C15—O1	0.2 (2)
N1—C7—C9—C10	176.07 (12)	C16—O2—C15—C11	179.37 (13)
C6—C7—C9—C10	−1.2 (2)	C12—C11—C15—O1	4.6 (2)
N2—C8—C9—C7	−0.63 (16)	C10—C11—C15—O1	−175.57 (14)
N2—C8—C9—C10	−177.05 (12)	C12—C11—C15—O2	−174.55 (12)
C7—C9—C10—C11	−95.43 (16)	C10—C11—C15—O2	5.31 (17)
C8—C9—C10—C11	80.01 (15)	C20—O4—C19—O3	−0.85 (19)
C7—C9—C10—C14	143.21 (14)	C20—O4—C19—C14	179.34 (11)
C8—C9—C10—C14	−41.35 (16)	C13—C14—C19—O3	9.1 (2)
C9—C10—C11—C12	−89.73 (14)	C10—C14—C19—O3	−174.67 (13)
C14—C10—C11—C12	31.92 (16)	C13—C14—C19—O4	−171.09 (12)
C9—C10—C11—C15	90.41 (14)	C10—C14—C19—O4	5.13 (16)

### Hydrogen-bond geometry (Å, º)

**Table:**

*D*—H···*A*	*D*—H	H···*A*	*D*···*A*	*D*—H···*A*
C18—H18*A*···O3	0.98	2.29	2.9114 (18)	121
N3—H1*N*3···N2^i^	0.93 (2)	2.14 (2)	3.0529 (17)	167 (2)
N1—H1*N*1···O3^ii^	0.84 (3)	2.01 (3)	2.8438 (16)	173 (2)
C5—H5*A*···O1^iii^	0.95	2.60	3.4895 (18)	157
C20—H20*C*···O1^iv^	0.98	2.38	3.3524 (19)	170

Symmetry codes: (i) −*x*+1, −*y*+2, *z*−1/2; (ii) −*x*+1/2, *y*−1/2, *z*+1/2; (iii) −*x*+1, −*y*+1, *z*+1/2; (iv) −*x*+1/2, *y*+1/2, *z*+1/2.
